# Cancer incidence among male construction workers in Korea: a standardized incidence ratio analysis, 2009-2015

**DOI:** 10.4178/epih.e2023060

**Published:** 2023-06-19

**Authors:** Soonsu Shin, Woo-Ri Lee, Jin-Ha Yoon, Wanhyung Lee

**Affiliations:** 1 Department of Preventive Medicine, Graduate School, Kyung Hee University, Seoul, Korea; 2 Department of Occupational and Environmental Medicine, Kyung Hee University Hospital, Seoul, Korea; 3 Division of Cancer Control & Policy, National Cancer Control Institute, National Cancer Center, Goyang, Korea; 4 Department of Preventive Medicine, Yonsei University College of Medicine, Seoul, Korea; 5 The Institute for Occupational Health, Yonsei University College of Medicine, Seoul, Korea; 6 Department of Occupational and Environmental Medicine, Gil Medical Center, Gachon University College of Medicine, Incheon, Korea

**Keywords:** Construction industry, Incidence, Neoplasm, Occupational groups

## Abstract

**OBJECTIVES:**

Construction workers face an elevated risk for several types of cancer. Nevertheless, there is a lack of large-scale epidemiological studies examining the risk of all cancers in construction workers. This study aimed to investigate the risk of various cancers in male construction workers using the Korean National Health Insurance Service (NHIS) database.

**METHODS:**

We used data from the NHIS database from 2009 to 2015. Construction workers were identified using the Korean Standard Industrial Classification code. We calculated the age-standardized incidence ratios (SIRs) and 95% confidence intervals (CIs) for cancer occurrence in male construction workers compared to all male workers.

**RESULTS:**

Compared to all male workers, the SIRs for esophageal cancer (SIR, 1.24; 95% CI, 1.07 to 1.42) and malignant neoplasms of the liver and intrahepatic bile ducts (SIR, 1.18; 95% CI, 1.13 to 1.24) were significantly higher in male construction workers. The SIRs for malignant neoplasms of the urinary tract (SIR, 1.19; 95% CI, 1.05 to 1.35) and non-Hodgkin lymphoma (SIR, 1.21; 95% CI, 1.02 to 1.43) were significantly elevated in building construction workers. The SIR for malignant neoplasms of the trachea, bronchus, and lung (SIR, 1.16; 95% CI, 1.03 to 1.29) was significantly higher in heavy and civil engineering workers.

**CONCLUSIONS:**

Male construction workers have an increased risk for esophageal cancer, liver cancer, lung cancer, and non-Hodgkin’s cancer. Our results indicate that tailored strategies for cancer prevention should be developed for construction workers.

## GRAPHICAL ABSTRACT

[Fig f2-epih-45-e2023060]

## INTRODUCTION

Occupational cancer is a serious health concern resulting from exposure to carcinogens in the workplace [1]. The International Agency for Research on Cancer (IARC) has classified 47 agents as “occupational carcinogens” among the 120 agents classified in IARC Group 1 (carcinogenic to humans), such as asbestos, silica dust, wood dust, and 1,3-butadiene [2]. Construction workers are exposed to various occupational hazards. Asbestos, for example, has been used widely as an insulation material at construction sites [3]. Asbestos is a major cause of mesothelioma, lung cancer, larynx cancer, and ovarian cancer [2]. Silica dust exposure is common among construction workers and causes lung cancer [2,4]. Carpenters and other woodworkers at construction sites are exposed to wood dust, which can cause nasopharynx cancer [2,5]. Additionally, construction workers who handle construction materials can be exposed to 1,3-butadiene, which has been linked to the development of leukemia [2,6].

Many efforts have been made to identify occupational cancers among construction workers. An increased risk of lung cancer and mesothelioma has been observed in this population [7,8]. Additionally, several studies have documented a heightened risk of gastric cancer in construction workers [9,10]. In a retrospective cohort study conducted in Sweden, researchers investigated the risk of various cancers among concrete workers [11]. The study reported a higher incidence of lip, stomach, and prostate cancers in male concrete workers [11].

In the United Kingdom, construction workers accounted for 41% of all occupational cancers in 2005, representing the highest incidence among all industrial groups [12]. However, between 2010 and 2016, the number of approved cases of occupational cancer among construction workers in Korea was relatively low, comprising only 7.7% of the total number of occupational cancers [13]. To the best of our knowledge, there have been few studies examining the risk of cancer among construction workers in Korea, although it has been reported that construction workers at petrochemical plants in Yeosu and Kwangyang faced an elevated risk of oral and pharyngeal cancer [14,15].

Numerous studies have been conducted on occupational cancer in construction workers, yet there remains a need to evaluate the current cancer risk in this profession, particularly in Korea. Prior research has not focused on the entire population of construction workers, but rather limited their scope to specific occupational groups within the construction industry. In this study, our aim was to investigate the risk of cancer among male construction workers using a large cohort based on the database of the Korean National Health Insurance Service (NHIS). Additionally, we divided construction workers into subgroups according to the Korean Standard Industrial Classification (KSIC) and calculated the age-standardized incidence ratios (SIRs) of cancer for each subgroup.

## MATERIALS AND METHODS

### Data

This study utilized the database of the NHIS. In Korea, all citizens are mandated to have health insurance through the NHIS, which operates under the laws governing long-term care, Medical Aid, and health insurance [16]. The NHIS database contains personal information of subscribers, such as identification number, age, sex, region, income, type of insurance, and industrial category. Furthermore, the database encompasses medical usage records of subscribers, including diagnoses, prescriptions, and procedures. Medical diagnoses were categorized according to the Korean Standard Classification of Diseases fourth edition, which corresponds to the International Classification of Diseases, 10th revision (ICD-10) [16].

### Study participants

Individuals insured under the NHIS are categorized into 2 types: the employee insured and the self-employed insured [16]. The employee insured group consists of wage workers and employers across all industries. In contrast, the self-employed insured group includes individuals who are not employees and their dependents, such as self-employed persons, farmers, and fishermen. For this study, we selected male employee insured individuals aged 25-64 years from NHIS subscribers in 2009. Their occupations were classified using the KSIC, established by Statistics Korea, following the fourth revision of the International Standard Industrial Classification of All Economic Activities (ISIC) in 2008 [17].

The ability of employees to change jobs, modify their working status, or transition in and out of workplaces during the follow-up period presents a significant challenge for occupation-based cohort studies. The NHIS updates subscribers’ data for occupations and industries annually, allowing us to track the working status and occupation of study participants on a yearly basis. We employed an open cohort design, defining construction workers as those who had worked in the construction industry at least once between 2009 and 2015. Male employee insured construction workers were identified as belonging to the group “F: Construction” according to KSIC at least once from 2009 to 2015. “F: Construction” is divided into seven subgroups: “building construction,” “heavy and civil engineering,” “site preparation and foundation,” “installing building equipment,” “electric and communication,” “building completion and finishing,” and “equipment rental and operational” [17].

We tracked the study participants from their enrollment year until 2015. Participants who developed cancer within 1 year of cohort entry were excluded from the study. Furthermore, construction workers who joined the cohort after 2013 were excluded, as they could not fulfill the minimum observation period of 2 years.

### Cancers

Cancer incidence was identified based on inpatient claims data with the ICD-10 code “C00-C97, malignant neoplasms” as the primary diagnosis. Malignant neoplasms were classified into 7 groups and 27 subgroups based on the Korean Standard Classification of Diseases, which codes according to the human organ system. The ICD-10 codes used to diagnose cancer were as follows: malignant neoplasm of the lip, oral cavity, and pharynx (C00-C14); esophagus (C15); stomach (C16); colon (C18); rectosigmoid junction, rectum, anus, and anal canal (C19-C21); liver and intrahepatic bile ducts (C22); pancreas (C25); other digestive organs (C17, C23, C24, C26); larynx (C32); trachea, bronchus, and lung (C33, C34); other respiratory and intrathoracic organs (C30, C31, C37-C39); bone and articular cartilage (C40, C41); malignant melanoma of the skin (C43); other skin (C44); mesothelial and soft tissue (C45-C49); prostate (C61); other male genital organs (C60, C62, C63); bladder (C67); other urinary tract (C64-C66, C68); eye and adnexa (C69); brain (C71); other parts of the central nervous system (C70, C72); Hodgkin’s disease (C81); non-Hodgkin lymphoma (C82-C86); leukemia (C91-C95); other lymphoid, hematopoietic, and related tissue (C88-C90, C96); and other, ill-defined, secondary, unspecified, and multiple sites (C73-C80, C97) [18].

### Statistical analysis

Cancer cases, SIRs, and 95% confidence intervals (CIs) were estimated among male construction workers. The reference population consisted of all male workers who did not work in construction during the follow-up period. We employed the indirect standardization method, and age-standardization was conducted using a 5-year standardization method for ages 25 years to 64 years old. To calculate SIRs, the number of observed cancer cases among construction workers was divided by the expected number of cancer cases. The expected number of cancer cases was determined by calculating the incidence rate of each cancer in the total male worker population. The 95% CIs were calculated based on the Poisson distribution, which is referred to as the “mid-P confidence interval” [19]. When both the SIR and the lower limit of the 95% CI were greater than 1.00, we considered this to indicate a statistically significant increase in cancer risk. All statistical analyses were performed using SAS version 9.4 (SAS Institute Inc., Cary, NC, USA) and R version 4.2.1 (R Foundation for Statistical Computing, Vienna, Austria).

### Ethics statement

Studies involving human participants were reviewed and approved by the Institutional Review Board of the Yonsei University Health System (IRB No. Y-2017-0100). The requirement for informed consent to participate was waived for this study because the data used in the present study were anonymized before their release from the NHIS.

## RESULTS

We observed 7,362,615 male workers and 598,155 male construction workers during the follow-up period (Table 1). Construction workers had a higher proportion of individuals aged 35 to 64 than the overall working population.

**Figure 1.**
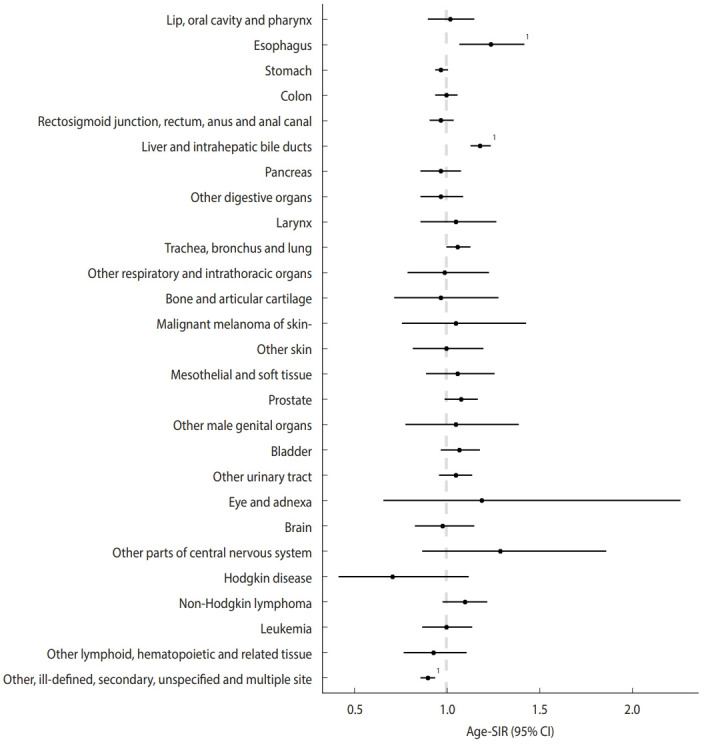
Age-standardized incidence ratios (SIRs) and 95% confidence intervals (CIs) of cancer types in male construction workers. ^1^CIs did not include 1.00.

Figure 1 and Table 2 display the SIRs and 95% CIs for each cancer type among male construction workers. The SIRs for esophageal cancer (SIR, 1.24; 95% CI, 1.07 to 1.42) and malignant neoplasms of the liver and intrahepatic bile ducts (SIR, 1.18; 95% CI, 1.13 to 1.24) were significantly higher for male construction workers compared to the total male worker population. However, the SIR for malignant neoplasms of other, ill-defined, secondary, unspecified, and multiple sites was significantly lower in male construction workers (SIR, 0.90; 95% CI, 0.86 to 0.94).

We calculated the SIRs and 95% CIs for cancer in 7 subgroups of male construction workers (Supplementary Materials 1-7). The SIR for esophageal cancer (SIR, 1.48; 95% CI, 1.01 to 2.10) was highest in site preparation and foundation workers (Supplementary Material 3). The SIR for malignant neoplasm of the liver and intrahepatic bile ducts (SIR, 1.36; 95% CI, 1.02 to 1.77) was highest in installing building equipment workers (Supplementary Material 4). The SIRs for malignant neoplasm of the urinary tract (SIR, 1.19; 95% CI, 1.05 to 1.35) and non-Hodgkin lymphoma (SIR, 1.21; 95% CI, 1.02 to 1.43) were significantly elevated in building construction workers (Supplementary Material 1). The SIR for malignant neoplasms of the trachea, bronchus, and lung (SIR, 1.16; 95% CI, 1.03 to 1.29) was significantly higher in heavy and civil engineering workers (Supplementary Material 2).

## DISCUSSION

In the present study, we assessed the risk of cancer among male construction workers. We found a significant increase in the risk of esophageal cancer and malignant neoplasms of the liver and intrahepatic bile ducts among construction workers in the Korea. In subgroup analyses, we observed that the cancer risk varied for each subgroup. This variation may be attributed to exposure to distinct health hazards, depending on the specific tasks performed by workers at the construction site.

Our findings regarding the elevated risk of esophageal cancer among construction workers align with those of prior research. Previous studies have indicated that construction workers face a heightened risk of esophageal cancer [20,21]. In an investigation involving 15 million workers across Nordic countries from 1960 to 1990, the SIR for esophageal cancer rose among male construction workers and painters [20]. Additionally, a case-control study carried out in 10 European countries revealed an increased OR for esophageal cancer in bricklayers [21].

Construction workers are exposed to external airborne agents (EAAs) that are present in the air within their workplace. EAAs, such as particulate matter (PM), mineral dust, silica dust, cement dust, and asbestos, are recognized as major risk factors for esophageal cancer [22]. A recent large-scale population study in China also found that environmental exposure to PM with diameters less than 2.5 μm (PM_2.5_) is associated with an increased incidence of esophageal cancer [23]. Similarly, site preparation and foundation workers, who are presumed to be exposed to large quantities of EAA, had the highest SIR for esophageal cancer in this study. Meyer et al. [24] suggested that the pulmonary clearance system removes inhaled EAAs from the respiratory tract, and the removed EAAs could then be swallowed by the digestive tract. EAAs have the potential to function as mutagens [25].

Night shift work and exposure to polycyclic aromatic hydrocarbons (PAHs) may contribute to the development of esophageal cancer. In 2007, the IARC classified night shift work as “probably carcinogenic to humans” (Group 2A) [26]. Among construction workers in the United States, 1.8% worked between 1:00 and 5:00 more than 5 times per month [26]. A cohort study conducted on the Japanese population found that male workers in rotating shifts had a higher risk of esophageal cancer [27]. Construction workers are exposed to PAHs through the incomplete combustion of diesel and gasoline [28]. Gustavsson et al. [29] observed an increased SIR of esophageal cancer among chimney sweeps and suggested that PAH exposure may be the cause. In regions with high PAH exposure, there is a high incidence rate of esophageal squamous cell carcinoma [30]. A recent in silico study reported that benzopyrene has a high affinity for Toll-like receptor 4, which is present in human esophageal epithelial cells, and may cause esophageal cancer [31].

In the subgroup analysis, the SIR for lung cancer was found to be increased only in heavy and civil engineering workers compared to the total male worker population. Previous studies have well-established that construction workers are at a higher risk of developing lung cancer [7,8,20]. Heavy and civil engineering workers are exposed to various types of dust, including silica and wood dust, which have been linked to lung cancer [2,4,5]. Experimental evidence from rodent models has shown that the intra-pulmonary administration of silica and wood dust triggers inflammatory responses in lung tissues [32,33]. Chronic inflammation has been demonstrated to contribute to the development of lung cancer [34].

This study discovered a heightened SIR for liver cancer in male construction workers. Previous research found no significant difference in liver cancer incidence among construction workers [20,35]. However, the same study revealed an increased risk of liver cancer in workers exposed to inorganic dust [35]. In Korea, construction sites have recently been identified as major sources of PM with diameters less than 10 μm (PM_10_) or PM_2.5_ [36].

Consequently, Korean construction workers are exposed to higher levels of PM_10_ and PM_2.5_ compared to the general population. Increased levels of PM_2.5_ have been associated with a higher risk of liver cancer [37]. Long-term exposure to PM_2.5_ has been shown to cause oxidative stress in hepatocytes [38]. Oxidative stress, which impairs DNA repair function, has been connected to the development of liver cancer [39].

According to our findings, construction workers involved in building construction have a significantly elevated risk of nonHodgkin lymphoma and malignant neoplasms of the urinary tract. However, prior cohort studies have not identified an increased risk of non-Hodgkin lymphoma among construction workers [14,24,40]. Construction workers may be exposed to organic solvents, such as primers, thinners, adhesives, and paints, during their work. These organic solvents contain various volatile organic compounds, including benzene, toluene, ethylbenzene, and xylene [41]. Population-based case-control studies have suggested that occupational exposure to organic solvents is associated with an increased risk of non-Hodgkin lymphoma [42,43]. A recent meta-analysis demonstrated that individuals with high benzene exposure had a greater relative risk of developing diffuse large B-cell lymphoma, a subtype of non-Hodgkin lymphoma [44].

Our research offers comprehensive information that can serve as a foundation for occupational safety and health policies aimed at preventing cancer among construction workers in Korea. In comparison to Canada and Europe, the number of claims for occupational cancer in the Korean construction industry is significantly lower [13]. However, this study identified higher SIRs for esophageal, liver, lung, and non-Hodgkin lymphoma cancers. This highlights the importance of implementing education, promotion, and prevention policies targeting occupational cancer among construction workers. Although our results did not show statistically significant findings for some types of cancer, it would be premature to conclude that construction workers are not at risk for these cancers. This is because we did not estimate the risk of cancer at the individual level, taking into account the exposure level of occupational hazards. The occupational hazards that construction workers face can vary based on the duration and specifics of their work processes at the construction site [45]. Considering the variability of occupational hazards depending on the duration and specific work processes at construction sites, there is a need to develop guidelines for measuring occupational hazards [45].

This is the first study to investigate the risk of all cancers in male construction workers. We examined over 600,000 male construction workers, which lends strength to our study due to its large sample size. Our dynamic cohort design offers the advantage of enrolling more subjects, as it includes workers who enrolled at least once during the cohort follow-up period. This approach also allows day laborers and temporary workers to be classified as construction workers. In 2014, 62.7% of construction employees in the Korea had precarious employment [46]. Moreover, since this study encompassed workers from 2009 to 2015, it reflects the current workplace environment of construction sites. Additionally, we calculated the SIRs of cancer for male construction workers categorized into subgroups based on standardized industry classification.

However, this study has several limitations. First, the hazard ratio was not determined after adjusting for past medical history and lifestyle behaviors. Factors such as smoking and drinking, which are known to be major causes of cancer, were not considered. The population-attributable fraction of smoking and alcohol consumption among Korean male was 20.9% and 3.0%, respectively, for the incidence of all cancers [47,48]. Therefore, the results of this study should be interpreted only as indicating a high incidence of certain cancers in male construction workers. Nevertheless, the findings could draw the attention of policymakers and administrators to the health of construction workers. Second, our results should not be extended to the individual level, as we adopted an ecological study design. Third, the latent period of occupational cancer was not considered. The duration of time between the initial exposure to a carcinogen and the cancer diagnosis is known as the latent period for cancer. For example, it has been estimated that the latent period for asbestos-related lung cancer and malignant mesothelioma is about 30-40 years [49]. Occupational cancers with a long latent period might not have been discovered during our follow-up period. Fourth, we identified cancer in the study using the ICD-10 code rather than a histological diagnosis. Fifth, it is possible that the healthy worker effect may have underestimated the risk of cancer among construction workers. To minimize the healthy worker effect, the current study used all male workers as the reference group. However, the overall healthy worker effect tends to be stronger among blue-collar workers who perform physically demanding labor [50].

In conclusion, male construction workers exhibited a relatively higher incidence of esophageal cancer and malignant neoplasms of the liver and intrahepatic bile ducts compared to the total male worker population. Additionally, this study revealed that cancer incidence varied among specific job roles within the construction industry, as demonstrated in the subgroup analysis. We anticipate that this research will serve as a foundation for future studies investigating occupational carcinogens among construction workers. To identify which occupational hazards contribute to cancer development, further research employing job exposure matrices is necessary.

## DATA AVAILABILITY

The datasets were not publicly available because the data belonged to the Korean government.

## Figures and Tables

**Figure f2-epih-45-e2023060:**
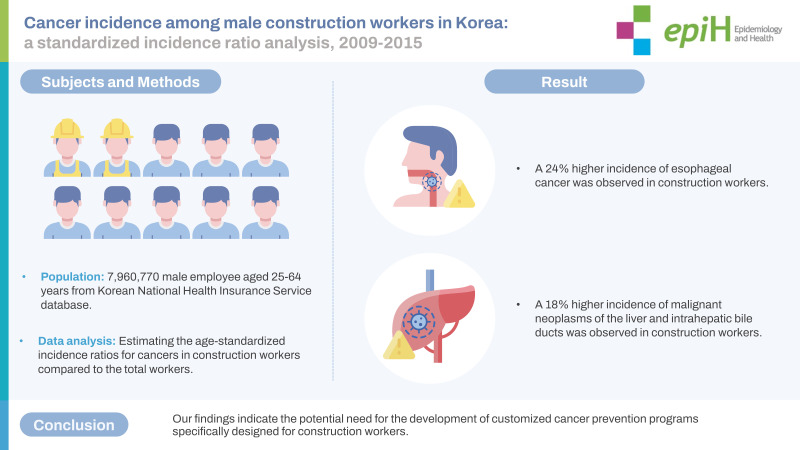


**Table 1.** Descriptive statistics of the study participants

Variables	Male construction workers	All male workers
Total (n)	598,155	7,362,615
Follow-up, mean±SD (yr)	6.72±1.08	6.80±0.90
Age (yr)		
25-29	51,415 (8.6)	960,520 (13.0)
30-34	85,008 (14.2)	1,243,816 (16.9)
35-39	115,488 (19.3)	1,354,220 (18.4)
40-44	108,184 (18.1)	1,174,744 (16.0)
45-49	92,386 (15.4)	1,040,067(14.1)
50-54	73,898 (12.3)	825,537 (11.2)
55-59	44,315 (7.4)	483,922 (6.6)
60-64	27,461 (4.6)	279,789 (3.8)

Values are presented as number (%).

**Table 2.** Age-standardized incidence ratios (SIRs) and 95% confidence intervals (CIs) for cancers in male construction workers compared to all male workers

ICD-10		Cancers	Expected cases	Observed cases	SIR (95% CI)
Gastrointestinal system					
	C00-C14	Malignant neoplasm of lip, oral cavity, and pharynx	256.47	262	1.02 (0.90, 1.15)
	C15	Malignant neoplasm of esophagus	157.61	195	1.24 (1.07, 1.42)
	C16	Malignant neoplasm of stomach	2,469.99	2,405	0.97 (0.94, 1.01)
	C18	Malignant neoplasm of colon	990.06	987	1.00 (0.94, 1.06)
	C19-C21	Malignant neoplasm of rectosigmoid junction, rectum, anus, and anal canal	872.44	849	0.97 (0.91, 1.04)
	C22	Malignant neoplasm of liver and intrahepatic bile ducts	1,518.18	1,792	1.18 (1.13, 1.24)
	C25	Malignant neoplasm of pancreas	301.24	291	0.97 (0.86, 1.08)
	C17, C23-C24, C26	Other malignant neoplasm of digestive organs	299.36	291	0.97 (0.86, 1.09)
Respiratory system					
	C32	Malignant neoplasm of larynx	99.33	104	1.05 (0.86, 1.27)
	C33-34	Malignant neoplasm of trachea, bronchus, and lung	1,083.71	1,151	1.06 (1.00, 1.13)
	C30-C31, C37-C39	Other malignant neoplasm of respiratory and intrathoracic organs	78.78	77	0.99 (0.79, 1.23)
Bone and skin					
	C40-C41	Malignant neoplasm of bone and articular cartilage	51.59	50	0.97 (0.72, 1.28)
	C43	Malignant melanoma of skin	38.86	41	1.05 (0.76, 1.43)
	C44	Other malignant neoplasm of skin	112.02	112	1.00 (0.82, 1.20)
	C45-C49	Malignant neoplasm of mesothelial and soft tissue	121.45	23	1.06 (0.89, 1.26)
Male reproductive system					
	C61	Malignant neoplasm of prostate	645.72	574	0.89 (0.82, 0.96)
	C60, C62-C63	Other malignant neoplasm of male genital organs	47.51	50	1.05 (0.78, 1.39)
Urinary system					
	C67	Malignant neoplasm of bladder	369.02	395	1.07 (0.97, 1.18)
	C64-C66, C68	Other malignant neoplasm of urinary tract	554.04	580	1.05 (0.96, 1.14)
Nervous system					
	C69	Malignant neoplasm of eye and adnexa	7.55	9	1.19 (0.55, 2.26)
	C71	Malignant neoplasm of brain	149.73	147	0.98 (0.83, 1.15)
	C70, 72	Malignant neoplasm of other parts of central nervous system	22.40	29	1.29 (0.87, 1.86)
Lymphoid and hematopoietic system					
		Hodgkin disease	25.50	18	0.71 (0.42, 1.12)
	C82-C86	Non-Hodgkin lymphoma	308.32	338	1.10 (0.98, 1.22)
	C91-C95	Leukemia	210.16	220	1.00 (0.87, 1.14)
	C88-C90, C96	Other malignant neoplasm of lymphoid, hematopoietic and related tissue	128.49	119	0.93 (0.77, 1.11)
Other					
	C73-C80, C97	Malignant neoplasm of other, ill-defined, secondary, unspecified, and multiple sites	2,636.64	2,374	0.90 (0.86, 0.94)

ICD-10, International Classification of Diseases, 10th revision.

## SUPPLEMENTARY MATERIALS

Supplementary Material 1. Age-standardized incidence ratios (SIRs) and 95% confidence intervals (CI) for cancers of building construction workers compared to total male workers

Supplementary Material 2 Age–standardized incidence ratios (SIRs) and 95% confidence intervals (CI) for cancers in heavy and civil engineering workers compared to total male workers

Supplementary Material 3 Age–standardized incidence ratios (SIRs) and 95% confidence intervals (CI) for cancers in site preparation and foundation workers compared to total male workers

Supplementary Material 4 Age–standardized incidence ratios (SIRs) and 95% confidence intervals (CI) for cancers in installing building equipment workers compared to total male workers

Supplementary Material 5 Age–standardized incidence ratios (SIRs) and 95% confidence intervals (CI) for cancers in the electric and communication workers compared to the total number of male workers

Supplementary Material 6 Age–standardized incidence ratios (SIRs) and 95% confidence intervals (CI) for cancers in building completion and finishing workers compared to total male workers

Supplementary Material 7 Age–standardized incidence ratios (SIRs) and 95% confidence intervals (CI) for cancers in equipment rental and operational workers compared to total male workers
