# Supplementary material for: Cancer incidence among male construction workers in Korea: a standardized incidence ratio analysis, 2009-2015
Source: Epidemiol Health. 2023 Jun 19;45:e2023060. doi: 10.4178/epih.e2023060 (PMC10482566; doi:10.4178/epih.e2023060)
Supplement: Supplementary Material 1. — Age-standardized incidence ratios (SIRs) and 95% confidence intervals (CI) for cancers of building construction workers compared to total male workers [file epih-45-e2023060-Supplementary-1.docx]

**Supplementary Material 1.** Age-standardized incidence ratios (SIRs) and 95% confidence intervals (CI) for cancers of building construction workers compared to total male workers

| ICD-10 | Cancers | Expected cases | Observed cases | SIRs (95% CI) |
| --- | --- | --- | --- | --- |
| **Gastrointestinal system** | |  |  |  |
| C00-C14 | Malignant neoplasm of lip, oral cavity, and pharynx | 96.41 | 85 | 1.02 (0.70–1.09) |
| C15 | Malignant neoplasm of esophagus | 59.18 | 61 | 1.03 (0.79-1.32) |
| C16 | Malignant neoplasm of stomach | 926.00 | 866 | 0.94 (0.87–1.00) |
| C18 | Malignant neoplasm of colon | 371.90 | 360 | 0.97 (0.87–1.07) |
| C19-C21 | Malignant neoplasm of rectosigmoid junction, rectum, anus, and anal canal | 327.50 | 334 | 1.02 (0.91–1.14) |
| C22 | Malignant neoplasm of liver and intrahepatic bile ducts | 566.30 | 605 | 1.07 (0.98–1.16) |
| C25 | Malignant neoplasm of pancreas | 113.09 | 95 | 0.84 (0.68–1.03) |
| C17, C23-C24, C26 | Other malignant neoplasm of digestive organs | 112.50 | 112 | 1.00 (0.82–1.20) |
| **Respiratory system** | |  |  |  |
| C32 | Malignant neoplasm of larynx | 37.34 | 38 | 1.02 (0.72–1.40) |
| C33-34 | Malignant neoplasm of trachea, bronchus, and lung | 408.30 | 414 | 1.01 (0.92–1.12) |
| C30-C31, C37-C39 | Other malignant neoplasm of respiratory and intrathoracic organs | 29.92 | 26 | 0.87 (0.57–1.27) |
| **Bone and skin** | |  |  |  |
| C40-C41 | Malignant neoplasm of bone and articular cartilage | 19.78 | 16 | 0.81 (0.46–1.31) |
| C43 | Malignant melanoma of skin | 14.70 | 13 | 0.88 (0.47–1.51) |
| C44 | Other malignant neoplasm of skin | 42.31 | 51 | 1.21 (0.90–1.58) |
| C45-C49 | Malignant neoplasm of mesothelial and soft tissue | 46.10 | 49 | 1.06 (0.79–1.41) |
| **Male reproductive system** | |  |  |  |
| C61 | Malignant neoplasm of prostate | 244.10 | 245 | 1.00 (0.88–1.14) |
| C60, C62-C63 | Other malignant neoplasm of male genital organs | 18.74 | 14 | 0.75 (0.41–1.25) |
| **Urinary system** | |  |  |  |
| C67 | Malignant neoplasm of bladder | 139.00 | 147 | 1.06 (0.89–1.24) |
| C64–C66, C68 | Other malignant neoplasm of urinary tract | 208.75 | 249 | **1.19 (1.05–1.35)** |
| **Nervous system** | |  |  |  |
| C69 | Malignant neoplasm of eye and adnexa | 2.82 | 7 | 1.19 (0.55–2.26) |
| C71 | Malignant neoplasm of brain | 57.03 | 52 | 0.91 (0.68–1.20) |
| C70, 72 | Malignant neoplasm of other parts of central nervous system | 8.55 | 6 | 0.70 (0.26–1.53) |
| **Lymphoid and hematopoietic system** | |  |  |  |
| C81 | Hodgkin disease | 9.76 | 6 | 0.62 (0.23–1.34) |
| C82-C86 | Non-Hodgkin lymphoma | 116.69 | 141 | **1.21 (1.02–1.43)** |
| C91-C95 | Leukemia | 80.24 | 91 | 1.13 (0.91–1.39) |
| C88-C90, C96 | Other malignant neoplasm of lymphoid, hematopoietic and related tissue | 48.35 | 45 | 0.93 (0.68–1.25) |
| **Other** | |  |  |  |
| C73-C80, C97 | Malignant neoplasm of other, ill-defined, secondary, unspecified, and multiple sites | 1,003.80 | 1,072 | 1.07 (1.00–1.13) |
