# Supplementary material for: Cancer incidence among male construction workers in Korea: a standardized incidence ratio analysis, 2009-2015
Source: Epidemiol Health. 2023 Jun 19;45:e2023060. doi: 10.4178/epih.e2023060 (PMC10482566; doi:10.4178/epih.e2023060)
Supplement: Supplementary Material 2 — Age–standardized incidence ratios (SIRs) and 95% confidence intervals (CI) for cancers in heavy and civil engineering workers compared to total male workers [file epih-45-e2023060-Supplementary-2.docx]

**Supplementary Material 2.** Age–standardized incidence ratios (SIRs) and 95% confidence intervals (CI) for cancers in heavy and civil engineering workers compared to total male workers

| ICD-10 | Cancers | Expected cases | Observed cases | SIRs (95% CI) |
| --- | --- | --- | --- | --- |
| **Gastrointestinal system** | |  |  |  |
| C00-C14 | Malignant neoplasm of lip, oral cavity, and pharynx | 62.94 | 64 | 1.02 (0.78–1.30) |
| C15 | Malignant neoplasm of esophagus | 39.44 | 55 | **1.39 (1.05–1.82)** |
| C16 | Malignant neoplasm of stomach | 610.90 | 592 | 0.97 (0.89–1.05) |
| C18 | Malignant neoplasm of colon | 245.50 | 260 | 1.06 (0.93–1.20) |
| C19-C21 | Malignant neoplasm of rectosigmoid junction, rectum, anus, and anal canal | 215.40 | 203 | 0.94 (0.82–1.08) |
| C22 | Malignant neoplasm of liver and intrahepatic bile ducts | 375.60 | 447 | **1.19 (1.08–1.31)** |
| C25 | Malignant neoplasm of pancreas | 74.87 | 86 | 1.15 (0.92–1.42) |
| C17, C23-C24, C26 | Other malignant neoplasm of digestive organs | 74.44 | 70 | 0.94 (0.73–1.19) |
| **Respiratory system** | |  |  |  |
| C32 | Malignant neoplasm of larynx | 24.83 | 24 | 0.97 (0.62–1.44) |
| C33-34 | Malignant neoplasm of trachea, bronchus, and lung | 270.30 | 313 | **1.16 (1.03–1.29)** |
| C30-C31, C37-C39 | Other malignant neoplasm of respiratory and intrathoracic organs | 19.10 | 21 | 1.10 (0.68–1.68) |
| **Bone and skin** | |  |  |  |
| C40-C41 | Malignant neoplasm of bone and articular cartilage | 12.33 | 15 | 1.22 (0.68–2.01) |
| C43 | Malignant melanoma of skin | 9.47 | 9 | 0.95 (0.43–1.80) |
| C44 | Other malignant neoplasm of skin | 27.47 | 14 | 0.51 (0.28–0.85) |
| C45-C49 | Malignant neoplasm of mesothelial and soft tissue | 29.37 | 35 | 1.19 (0.83–1.66) |
| **Male reproductive system** | |  |  |  |
| C61 | Malignant neoplasm of prostate | 162.50 | 149 | 0.92 (0.78–1.08) |
| C60, C62-C63 | Other malignant neoplasm of male genital organs | 10.90 | 12 | 1.10 (0.57–1.92) |
| **Urinary system** | |  |  |  |
| C67 | Malignant neoplasm of bladder | 91.56 | 102 | 1.11 (0.91–1.35) |
| C64-C66, C68 | Other malignant neoplasm of urinary tract | 135.48 | 151 | 1.11 (0.94–1.31) |
| **Nervous system** | |  |  |  |
| C69 | Malignant neoplasm of eye and adnexa | 1.85 | 1 | 0.54 (0.01–3.02) |
| C71 | Malignant neoplasm of brain | 36.10 | 35 | 0.97 (0.68–1.35) |
| C70, 72 | Malignant neoplasm of other parts of central nervous system | 5.37 | 11 | **2.05 (1.02–3.66)** |
| **Lymphoid and hematopoietic system** | |  |  |  |
| C81 | Hodgkin disease | 6.07 | 3 | 0.49 (0.10–1.44) |
| C82-C86 | Non-Hodgkin lymphoma | 75.00 | 78 | 1.04 (0.82–1.30) |
| C91-C95 | Leukemia | 50.50 | 41 | 0.81 (0.58–1.10) |
| C88-C90, C96 | Other malignant neoplasm of lymphoid, hematopoietic and related tissue | 31.62 | 33 | 1.04 (0.72–1.47) |
| **Other** | |  |  |  |
| C73-C80, C97 | Malignant neoplasm of other, ill-defined, secondary, unspecified, and multiple sites | 632.20 | 551 | **0.87 (0.80–0.95)** |
