# Supplementary material for: Cancer incidence among male construction workers in Korea: a standardized incidence ratio analysis, 2009-2015
Source: Epidemiol Health. 2023 Jun 19;45:e2023060. doi: 10.4178/epih.e2023060 (PMC10482566; doi:10.4178/epih.e2023060)
Supplement: Supplementary Material 3 — Age–standardized incidence ratios (SIRs) and 95% confidence intervals (CI) for cancers in site preparation and foundation workers compared to total male workers [file epih-45-e2023060-Supplementary-3.docx]

**Supplementary Material 3**. Age–standardized incidence ratios (SIRs) and 95% confidence intervals (CI) for cancers in site preparation and foundation workers compared to total male workers

| ICD-10 | Cancers | Expected cases | Observed cases | SIRs (95% CI) |
| --- | --- | --- | --- | --- |
| **Gastrointestinal system** | |  |  |  |
| C00-C14 | Malignant neoplasm of lip, oral cavity, and pharynx | 33.88 | 37 | 1.09 (0.77–1.51) |
| C15 | Malignant neoplasm of esophagus | 20.96 | 31 | **1.48 (1.01–2.10)** |
| C16 | Malignant neoplasm of stomach | 328.20 | 318 | 0.97 (0.87–1.08) |
| C18 | Malignant neoplasm of colon | 131.20 | 124 | 0.95 (0.79–1.13) |
| C19-C21 | Malignant neoplasm of rectosigmoid junction, rectum, anus, and anal canal | 115.70 | 99 | 0.86 (0.70–1.04) |
| C22 | Malignant neoplasm of liver and intrahepatic bile ducts | 203.70 | 263 | **1.29 (1.14–1.46)** |
| C25 | Malignant neoplasm of pancreas | 39.95 | 38 | 0.95 (0.67–1.31) |
| C17, C23-C24, C26 | Other malignant neoplasm of digestive organs | 39.62 | 28 | 0.71 (0.47–1.02) |
| **Respiratory system** | |  |  |  |
| C32 | Malignant neoplasm of larynx | 13.16 | 17 | 1.29 (0.75–2.07) |
| C33-34 | Malignant neoplasm of trachea, bronchus, and lung | 143.00 | 147 | 1.03 (0.87–1.21) |
| C30-C31, C37-C39 | Other malignant neoplasm of respiratory and intrathoracic organs | 10.21 | 18 | **1.76 (1.04–2.79)** |
| **Bone and skin** | |  |  |  |
| C40-C41 | Malignant neoplasm of bone and articular cartilage | 6.57 | 11 | 1.67 (0.84–3.00) |
| C43 | Malignant melanoma of skin | 5.08 | 4 | 0.79 (0.21–2.02) |
| C44 | Other malignant neoplasm of skin | 14.68 | 17 | 1.16 (0.67–1.85) |
| C45-C49 | Malignant neoplasm of mesothelial and soft tissue | 15.75 | 18 | 1.15 (0.68–1.81) |
| **Male reproductive system** | |  |  |  |
| C61 | Malignant neoplasm of prostate | 84.79 | 64 | 0.93 (0.72–1.19) |
| C60, C62-C63 | Other malignant neoplasm of male genital organs | 5.73 | 9 | 1.57 (0.72–2.98) |
| **Urinary system** | |  |  |  |
| C67 | Malignant neoplasm of bladder | 48.67 | 44 | 0.90 (0.66–1.21) |
| C64-C66, C68 | Other malignant neoplasm of urinary tract | 72.89 | 69 | 0.95 (0.74–1.20) |
| **Nervous system** | |  |  |  |
| C69 | Malignant neoplasm of eye and adnexa | 1.00 | 0 | 0.00 (0.00–0.00) |
| C71 | Malignant neoplasm of brain | 19.28 | 20 | 1.04 (0.63–1.60) |
| C70, 72 | Malignant neoplasm of other parts of central nervous system | 2.87 | 4 | 1.39 (0.38–3.56) |
| **Lymphoid and hematopoietic system** | |  |  |  |
| C81 | Hodgkin disease | 3.26 | 3 | 0.92 (0.19–2.09) |
| C82-C86 | Non-Hodgkin lymphoma | 40.22 | 39 | 0.97 (0.69–1.33) |
| C91-C95 | Leukemia | 26.96 | 19 | 0.70 (0.42–1.10) |
| C88-C90, C96 | Other malignant neoplasm of lymphoid, hematopoietic and related tissue | 16.97 | 10 | 0.59 (0.28–1.08) |
| **Other** | |  |  |  |
| C73-C80, C97 | Malignant neoplasm of other, ill-defined, secondary, unspecified, and multiple sites | 339.10 | 249 | **0.73 (0.65–0.83)** |
