# Supplementary material for: Cancer incidence among male construction workers in Korea: a standardized incidence ratio analysis, 2009-2015
Source: Epidemiol Health. 2023 Jun 19;45:e2023060. doi: 10.4178/epih.e2023060 (PMC10482566; doi:10.4178/epih.e2023060)
Supplement: Supplementary Material 4 — Age–standardized incidence ratios (SIRs) and 95% confidence intervals (CI) for cancers in installing building equipment workers compared to total male workers [file epih-45-e2023060-Supplementary-4.docx]

**Supplementary Material 4**. Age–standardized incidence ratios (SIRs) and 95% confidence intervals (CI) for cancers in installing building equipment workers compared to total male workers

| ICD-10 | Cancers | Expected cases | Observed cases | SIRs (95% CI) |
| --- | --- | --- | --- | --- |
| **Gastrointestinal system** | |  |  |  |
| C00-C14 | Malignant neoplasm of lip, oral cavity, and pharynx | 6.72 | 8 | 1.19 (0.51–2.35) |
| C15 | Malignant neoplasm of esophagus | 4.24 | 6 | 1.42 (0.52–3.08) |
| C16 | Malignant neoplasm of stomach | 64.84 | 53 | 0.82 (0.62–1.07) |
| C18 | Malignant neoplasm of colon | 26.21 | 23 | 0.88 (0.56–1.32) |
| C19-C21 | Malignant neoplasm of rectosigmoid junction, rectum, anus, and anal canal | 22.94 | 23 | 1.00 (0.64–1.50) |
| C22 | Malignant neoplasm of liver and intrahepatic bile ducts | 39.72 | 54 | **1.36 (1.02–1.77)** |
| C25 | Malignant neoplasm of pancreas | 7.97 | 7 | 0.88 (0.35–1.81) |
| C17, C23-C24, C26 | Other malignant neoplasm of digestive organs | 7.95 | 11 | 1.38 (0.69–2.48) |
| **Respiratory system** | |  |  |  |
| C32 | Malignant neoplasm of larynx | 2.66 | 2 | 0.75 (0.09–2.71) |
| C33-34 | Malignant neoplasm of trachea, bronchus, and lung | 28.92 | 28 | 0.97 (0.64–1.40) |
| C30-C31, C37-C39 | Other malignant neoplasm of respiratory and intrathoracic organs | 2.06 | 0 | 0.00 (0.00–0.00) |
| **Bone and skin** | |  |  |  |
| C40-C41 | Malignant neoplasm of bone and articular cartilage | 1.34 | 0 | 0.00 (0.00–0.00) |
| C43 | Malignant melanoma of skin | 1.02 | 2 | 1.97 (0.24–7.10) |
| C44 | Other malignant neoplasm of skin | 2.95 | 5 | 1.70 (0.55–3.96) |
| C45-C49 | Malignant neoplasm of mesothelial and soft tissue | 3.18 | 2 | 0.63 (0.08–2.27) |
| **Male reproductive system** | |  |  |  |
| C61 | Malignant neoplasm of prostate | 17.52 | 15 | 0.86 (0.48–1.41) |
| C60, C62-C63 | Other malignant neoplasm of male genital organs | 1.26 | 1 | 0.79 (0.02–4.42) |
| **Urinary system** | |  |  |  |
| C67 | Malignant neoplasm of bladder | 9.80 | 9 | 0.92 (0.42–1.74) |
| C64-C66, C68 | Other malignant neoplasm of urinary tract | 14.46 | 8 | 0.55 (0.24–1.09) |
| **Nervous system** | |  |  |  |
| C69 | Malignant neoplasm of eye and adnexa | 0.20 | 0 | 0.00 (0.00–0.00) |
| C71 | Malignant neoplasm of brain | 3.91 | 1 | 0.26 (0.01–1.43) |
| C70, 72 | Malignant neoplasm of other parts of central nervous system | 0.58 | 0 | 0.00 (0.00–0.00) |
| **Lymphoid and hematopoietic system** | |  |  |  |
| C81 | Hodgkin disease | 0.67 | 0 | 0.00 (0.00–0.00) |
| C82-C86 | Non-Hodgkin lymphoma | 8.05 | 3 | 0.37 (0.08–1.09) |
| C91-C95 | Leukemia | 5.48 | 2 | 0.36 (0.04–1.32) |
| C88-C90, C96 | Other malignant neoplasm of lymphoid, hematopoietic and related tissue | 3.38 | 4 | 1.18 (0.32–3.03) |
| **Other** | |  |  |  |
| C73-C80, C97 | Malignant neoplasm of other, ill-defined, secondary, unspecified, and multiple sites | 68.30 | 59 | 0.86 (0.66–1.11) |
