# Supplementary material for: Cancer incidence among male construction workers in Korea: a standardized incidence ratio analysis, 2009-2015
Source: Epidemiol Health. 2023 Jun 19;45:e2023060. doi: 10.4178/epih.e2023060 (PMC10482566; doi:10.4178/epih.e2023060)
Supplement: Supplementary Material 5 — Age–standardized incidence ratios (SIRs) and 95% confidence intervals (CI) for cancers in the electric and communication workers compared to the total number of male workers [file epih-45-e2023060-Supplementary-5.docx]

**Supplementary Material 5.** Age–standardized incidence ratios (SIRs) and 95% confidence intervals (CI) for cancers in the electric and communication workers compared to the total number of male workers

| ICD-10 | Cancers | Expected cases | Observed cases | SIRs (95% CI) |
| --- | --- | --- | --- | --- |
| **Gastrointestinal system** | |  |  |  |
| C00-C14 | Malignant neoplasm of lip, oral cavity, and pharynx | 24.54 | 32 | 1.30 (0.89–1.84) |
| C15 | Malignant neoplasm of esophagus | 14.40 | 20 | 1.39 (0.85–2.15) |
| C16 | Malignant neoplasm of stomach | 233.10 | 259 | 1.11 (0.98–1.25) |
| C18 | Malignant neoplasm of colon | 92.67 | 106 | 1.14 (0.94–1.38) |
| C19-C21 | Malignant neoplasm of rectosigmoid junction, rectum, anus, and anal canal | 82.47 | 91 | 1.10 (0.89–1.35) |
| C22 | Malignant neoplasm of liver and intrahepatic bile ducts | 143.40 | 179 | **1.25 (1.07–1.45)** |
| C25 | Malignant neoplasm of pancreas | 28.08 | 32 | 1.14 (0.78–1.61) |
| C17, C23-C24, C26 | Other malignant neoplasm of digestive organs | 27.86 | 34 | 1.22 (0.85–1.71) |
| **Respiratory system** | |  |  |  |
| C32 | Malignant neoplasm of larynx | 9.12 | 8 | 0.88 (0.38–1.73) |
| C33-34 | Malignant neoplasm of trachea, bronchus, and lung | 99.81 | 112 | 1.12 (0.92–1.35) |
| C30-C31, C37-C39 | Other malignant neoplasm of respiratory and intrathoracic organs | 7.67 | 5 | 0.65 (0.21–1.52) |
| **Bone and skin** | |  |  |  |
| C40-C41 | Malignant neoplasm of bone and articular cartilage | 5.13 | 3 | 0.59 (0.12–1.71) |
| C43 | Malignant melanoma of skin | 3.75 | 6 | 1.60 (0.59–3.48) |
| C44 | Other malignant neoplasm of skin | 10.70 | 6 | 0.56 (0.21–1.22) |
| C45-C49 | Malignant neoplasm of mesothelial and soft tissue | 11.90 | 12 | 1.01 (0.52–1.76) |
| **Male reproductive system** | |  |  |  |
| C61 | Malignant neoplasm of prostate | 58.11 | 44 | 0.76 (0.55–1.02) |
| C60, C62-C63 | Other malignant neoplasm of male genital organs | 4.96 | 9 | 1.81 (0.83–3.44) |
| **Urinary system** | |  |  |  |
| C67 | Malignant neoplasm of bladder | 34.44 | 42 | 1.22 (0.88–1.65) |
| C64-C66, C68 | Other malignant neoplasm of urinary tract | 53.35 | 36 | **0.67 (0.47–0.93)** |
| **Nervous system** | |  |  |  |
| C69 | Malignant neoplasm of eye and adnexa |  |  | 0.00 (0.00–0.00) |
| C71 | Malignant neoplasm of brain |  |  | 0.26 (0.01–1.43) |
| C70, 72 | Malignant neoplasm of other parts of central nervous system |  |  | 0.00 (0.00–0.00) |
| **Lymphoid and hematopoietic system** | |  |  |  |
| C81 | Hodgkin disease |  |  | 0.00 (0.00–0.00) |
| C82-C86 | Non-Hodgkin lymphoma |  |  | 0.37 (0.08–1.09) |
| C91-C95 | Leukemia |  |  | 0.36 (0.04–1.32) |
| C88-C90, C96 | Other malignant neoplasm of lymphoid, hematopoietic and related tissue |  |  | 1.18 (0.32–3.03) |
| **Other** | |  |  |  |
| C73-C80, C97 | Malignant neoplasm of other, ill-defined, secondary, unspecified, and multiple sites |  |  | 0.86 (0.66–1.11) |
