# Supplementary material for: Cancer incidence among male construction workers in Korea: a standardized incidence ratio analysis, 2009-2015
Source: Epidemiol Health. 2023 Jun 19;45:e2023060. doi: 10.4178/epih.e2023060 (PMC10482566; doi:10.4178/epih.e2023060)
Supplement: Supplementary Material 6 — Age–standardized incidence ratios (SIRs) and 95% confidence intervals (CI) for cancers in building completion and finishing workers compared to total male workers [file epih-45-e2023060-Supplementary-6.docx]

**Supplementary Material 6.** Age–standardized incidence ratios (SIRs) and 95% confidence intervals (CI) for cancers in building completion and finishing workers compared to total male workers

| ICD-10 | Cancers | Expected cases | Observed cases | SIRs (95% CI) |
| --- | --- | --- | --- | --- |
| **Gastrointestinal system** | |  |  |  |
| C00-C14 | Malignant neoplasm of lip, oral cavity, and pharynx | 26.43 | 33 | 1.25 (0.86–1.75) |
| C15 | Malignant neoplasm of esophagus | 15.08 | 19 | 1.20 (0.72–1.87) |
| C16 | Malignant neoplasm of stomach | 252.90 | 255 | 1.01 (0.89–1.14) |
| C18 | Malignant neoplasm of colon | 100.80 | 95 | 0.94 (0.76–1.15) |
| C19-C21 | Malignant neoplasm of rectosigmoid junction, rectum, anus, and anal canal | 89.37 | 77 | 0.86 (0.68–1.08) |
| C22 | Malignant neoplasm of liver and intrahepatic bile ducts | 156.20 | 209 | **1.34 (1.16–1.53)** |
| C25 | Malignant neoplasm of pancreas | 30.63 | 27 | 0.88 (0.58–1.28) |
| C17, C23-C24, C26 | Other malignant neoplasm of digestive organs | 30.37 | 29 | 0.96 (0.64–1.37) |
| **Respiratory system** | |  |  |  |
| C32 | Malignant neoplasm of larynx | 10.00 | 11 | 1.10 (0.55–1.97) |
| C33-34 | Malignant neoplasm of trachea, bronchus, and lung | 109.30 | 108 | 0.99 (0.81–1.19) |
| C30-C31, C37-C39 | Other malignant neoplasm of respiratory and intrathoracic organs | 8.15 | 5 | 0.61 (0.20–1.43) |
| **Bone and skin** | |  |  |  |
| C40-C41 | Malignant neoplasm of bone and articular cartilage | 5.36 | 5 | 0.93 (0.30–2.17) |
| C43 | Malignant melanoma of skin | 4.01 | 7 | 1.75 (0.70–3.60) |
| C44 | Other malignant neoplasm of skin | 11.49 | 18 | 1.57 (0.93–2.48) |
| C45-C49 | Malignant neoplasm of mesothelial and soft tissue | 17.58 | 11 | 0.87 (0.44–1.56) |
| **Male reproductive system** | |  |  |  |
| C61 | Malignant neoplasm of prostate | 64.13 | 45 | **0.70 (0.51–0.94)** |
| C60, C62-C63 | Other malignant neoplasm of male genital organs | 4.99 | 4 | 0.80 (0.22–2.05) |
| **Urinary system** | |  |  |  |
| C67 | Malignant neoplasm of bladder | 37.43 | 41 | 1.10 (0.79–1.49) |
| C64-C66, C68 | Other malignant neoplasm of urinary tract | 58.18 | 52 | 0.91 (0.68–1.19) |
| **Nervous system** | |  |  |  |
| C69 | Malignant neoplasm of eye and adnexa | 0.79 | 1 | 1.27 (0.03–7.08) |
| C71 | Malignant neoplasm of brain | 15.52 | 20 | 1.29 (0.79–1.99) |
| C70, 72 | Malignant neoplasm of other parts of central nervous system | 2.32 | 4 | 1.72 (0.47–4.41) |
| **Lymphoid and hematopoietic system** | |  |  |  |
| C81 | Hodgkin disease | 2.66 | 5 | 1.68 (0.61–4.38) |
| C82-C86 | Non-Hodgkin lymphoma | 31.87 | 28 | 0.88 (0.58–1.27) |
| C91-C95 | Leukemia | 21.82 | 23 | 1.05 (0.67–1.58) |
| C88-C90, C96 | Other malignant neoplasm of lymphoid, hematopoietic and related tissue | 13.18 | 15 | 1.14 (0.64–1.88) |
| **Other** | |  |  |  |
| C73-C80, C97 | Malignant neoplasm of other, ill-defined, secondary, unspecified, and multiple sites | 275.1 | 215 | **0.78 (0.68–0.89)** |
