# Supplementary material for: Cancer incidence among male construction workers in Korea: a standardized incidence ratio analysis, 2009-2015
Source: Epidemiol Health. 2023 Jun 19;45:e2023060. doi: 10.4178/epih.e2023060 (PMC10482566; doi:10.4178/epih.e2023060)
Supplement: Supplementary Material 7 — Age–standardized incidence ratios (SIRs) and 95% confidence intervals (CI) for cancers in equipment rental and operational workers compared to total male workers [file epih-45-e2023060-Supplementary-7.docx]

**Supplementary Material 7.** Age–standardized incidence ratios (SIRs) and 95% confidence intervals (CI) for cancers in equipment rental and operational workers compared to total male workers

| ICD-10 | Cancers | Expected cases | Observed cases | SIRs (95% CI) |
| --- | --- | --- | --- | --- |
| **Gastrointestinal system** | |  |  |  |
| C00-C14 | Malignant neoplasm of lip, oral cavity, and pharynx | 5.56 | 3 | 0.54 (0.11–1.58) |
| C15 | Malignant neoplasm of esophagus | 3.54 | 3 | 0.85 (0.17–2.48) |
| C16 | Malignant neoplasm of stomach | 56.06 | 62 | 1.15 (0.88–1.47) |
| C18 | Malignant neoplasm of colon | 21.78 | 19 | 0.87 (0.53–1.36) |
| C19-C21 | Malignant neoplasm of rectosigmoid junction, rectum, anus, and anal canal | 19.06 | 22 | 1.15 (0.72–1.75) |
| C22 | Malignant neoplasm of liver and intrahepatic bile ducts | 33.26 | 35 | 1.05 (0.73–1.46) |
| C25 | Malignant neoplasm of pancreas | 6.65 | 6 | 0.90 (0.33–1.97) |
| C17, C23-C24, C26 | Other malignant neoplasm of digestive organs | 6.61 | 7 | 1.06 (0.43–2.18) |
| **Respiratory system** | |  |  |  |
| C32 | Malignant neoplasm of larynx | 2.22 | 4 | 1.80 (0.49–4.61) |
| C33-34 | Malignant neoplasm of trachea, bronchus, and lung | 24.08 | 29 | 1.20 (0.81–1.73) |
| C30-C31, C37-C39 | Other malignant neoplasm of respiratory and intrathoracic organs | 1.68 | 2 | 1.19 (0.14–4.30) |
| **Bone and skin** | |  |  |  |
| C40-C41 | Malignant neoplasm of bone and articular cartilage | 1.07 | 0 | 0.00 (0.00–0.00) |
| C43 | Malignant melanoma of skin | 0.84 | 0 | 0.00 (0.00–0.00) |
| C44 | Other malignant neoplasm of skin | 2.43 | 1 | 0.41 (0.01–2.30) |
| C45-C49 | Malignant neoplasm of mesothelial and soft tissue | 2.57 | 2 | 0.78 (0.09–2.81) |
| **Male reproductive system** | |  |  |  |
| C61 | Malignant neoplasm of prostate | 14.57 | 12 | 0.82 (0.43–1.44) |
| C60, C62-C63 | Other malignant neoplasm of male genital organs | 0.93 | 1 | 1.07 (0.03–5.96) |
| **Urinary system** | |  |  |  |
| C67 | Malignant neoplasm of bladder | 8.13 | 10 | 1.23 (0.59–2.26) |
| C64-C66, C68 | Other malignant neoplasm of urinary tract | 11.94 | 15 | 1.26 (0.70–2.07) |
| **Nervous system** | |  |  |  |
| C69 | Malignant neoplasm of eye and adnexa | 0.16 | 0 | 0.00 (0.00–0.00) |
| C71 | Malignant neoplasm of brain | 3.16 | 1 | 0.32 (0.01–1.76) |
| C70, 72 | Malignant neoplasm of other parts of central nervous system | 0.47 | 0 | 0.00 (0.00–0.00) |
| **Lymphoid and hematopoietic system** | |  |  |  |
| C81 | Hodgkin disease | 0.52 | 0 | 0.00 (0.00–0.00) |
| C82-C86 | Non-Hodgkin lymphoma | 6.58 | 11 | 1.67 (0.83–2.99) |
| C91-C95 | Leukemia | 4.41 | 7 | 1.59 (0.64–3.27) |
| C88-C90, C96 | Other malignant neoplasm of lymphoid, hematopoietic and related tissue | 2.80 | 3 | 1.07 (0.22–3.14) |
| **Other** | |  |  |  |
| C73-C80, C97 | Malignant neoplasm of other, ill-defined, secondary, unspecified, and multiple sites | 55.14 | 43 | 0.78 (0.56–1.05) |
